# Clinical practice guidelines for psychiatrists: Indian Psychiatric Society guidelines *vs*. international guidelines: A critical appraisal

**DOI:** 10.4103/0019-5545.37670

**Published:** 2007

**Authors:** Dishanter Goel, Jitendra Kumar Trivedi

**Affiliations:** Department of Psychiatry, King George's Medical University, Lucknow, Uttar Pradesh, India

**Keywords:** APA guidelines, ECGS, IPS guidelines, PORT recommendations, TMAP recommendations

## Abstract

Various guidelines have been proposed to assist psychiatrists all over the world in making appropriate health-care decisions. Though the fundamental premises of all guidelines are the same, yet they differ in certain important aspects; this hampers the universality of these guidelines. There are many internationally accepted guidelines which are based on robust research; still they do not necessarily address the geographical and cultural differences. This necessitates the formulation of regional guidelines, which usually lack the background of robust regional research. The Indian Psychiatric Society (IPS) guidelines were also formulated to cater to the needs of the Indian population. It is now almost three years old, and it is high time it should be compared to the international guidelines, so as to appraise ourselves of the success or shortcomings of the guidelines. This article critically analyzes the IPS guidelines in comparison with the available international guidelines and schematically brings out the positive points, as well as the shortcomings, with the aim of further improvement in our indigenous guidelines.

Various clinical practice guidelines have been proposed, to assist practitioners in making appropriate health-care decisions by synthesizing the treatment literature into a usable form and facilitating the transfer of research into practice. Guidelines have also been used to control practice variation, to lower costs and to evaluate care processes.

As many guidelines have been formulated, there have been problems of clinician's acceptance of the guidelines, as they lack universality. Despite the presence of international guidelines like American Psychiatric Association (APA) guidelines,[1] Patient Outcome Research Team (PORT) recommendations,[2] Expert Consensus Guidelines Series (ECGS) recommendations,[3] Texas Medication Algorithm Project (TMAP),[4] a need for regional guidelines was always felt.. Indian Psychiatric Society task force on clinical practice guidelines for psychiatrists in India proposed clinical practice guidelines[5] for psychiatrists in India, based on the national workshop held at Jaipur in 2004.

Though the IPS guidelines were drafted with utmost care, a comparison with the available international guidelines is necessary to impart a universal appeal to the guidelines. Such a comparison can bring to the fore many hidden issues, as well as serve to lift the standards of the guidelines to international levels. The IPS guidelines were compared with the international guidelines on the degree of scientific rigor, comprehensiveness and clinical parameters and clinical applicability, with the belief that it will assist the decision-making process for practitioners and health-care organizations that are considering the implementation of guidelines and will inform the development of future guidelines.

## COMPARISON OF GUIDELINES

### Scientific background

Scientific background makes the most important pillar of any standard guidelines [Table 1]. Guidelines developed with a high degree of scientific rigor are likely to be valid, reliable and reproducible. Ideally, guideline developers should specify treatment questions, conduct an extensive structured literature search, assess the quality of the studies identified in the search, report how the studies synthesized the scientific evidence, make key data available for readers and label the strength of the evidence for each treatment recommendation. Moreover, the guidelines need to consider the studies conducted on the local population.

**Table 1:** A tabular comparison of international and Indian guidelines

Characteristics	American psychiatric association (APA) guidelines	Patient outcome research team (PORT) recommendations	Expert consensus guideline series recommendations	Texas medication algorithm project (TMAP)	Indian psychiatric society (IPS) guidelines
Scientific background					
Structured literature review	Yes	Yes	No	No	No
Structurally defined evidence base	Yes	Yes	No	No	No
Rated level of evidence for each recommendation	Yes	Yes	No	No	No
Development process and review	Defined; rigorous	Defined; rigorous	Not detailed	Not defined	Not defined
Comprehensiveness and clinical parameters					
Literature review	Extensive and referenced in the recommendations	Extensive but referenced only	No review	No review	Minimal review; regional review present but only referenced
Co-morbid conditions	Extensive	Moderate	Moderate	Minimal	Minimal
Cost and resource considerations	Minimal	Minimal; discuss cost of assertive community training and clozapine	Minimal; discuss methods to maintain cost-effectiveness	No	Minimal
Psychosocial and cultural considerations	Moderate	Minimal	Minimal	Minimal	Minimal
Strategies to switch antipsychotics	No	No	Present	Present	No
Special situations; suicidality, aggression, subtypes, pregnancy	Discussed	Discussed	Discussed	Suicidality not discussed	Not discussed
Dosage recommendations	Present	Present	Present	Present	Not given
Clinical applicability					
Format	Literature review	Numbered recommendations	Tables	Algorithms	Guideline and literature review
Ease of use	Difficult to use	Yes	Yes	Yes	Difficult to use
General outline of guideline	Arranged to a defined format	Properly arranged	No defined format; arranged properly	Algorithmic	Not arranged to a defined format; vague

The guidelines produced by APA and by PORT are the most scientifically rigorous of those we examined. The developers of these guidelines conducted extensive structured literature reviews and considered study design and quality in making their recommendations. They also coded each recommendation to indicate the strength of the supporting evidence and included a list of references. However, neither of the sets of developers published formal evidence tables, and only PORT specified how expert review was incorporated into the final guideline.

The developers of the Expert Consensus guidelines and of the TMAP algorithms conducted limited literature searches and did not code recommendations to indicate the level of supporting evidence. Indeed, the Expert Consensus guidelines were developed specifically to provide assistance to clinicians in areas in which scientific evidence was weak or lacking.

The IPS guidelines consist of a detailed literature review, which adds to their effectiveness. It also mentions an elaborate list of the Indian studies, but the influence of these studies on the final drafting of the guidelines is not clear; and at most places, the guidelines seem to be a direct adaptation of the Western literature. Moreover, the studies occur separately as a table or in the bibliography, without any mention in the text.

### Comprehensiveness

Comprehensive guidelines for the treatment of schizophrenia address more phases of the illness, discuss a wider variety of treatment modalities and make greater numbers of specific recommendations. They also have greater depth, with recommendations reflecting previous treatment trials and responses. Ideally, costs and resource allocation are also considered.

### Literature review

The APA guideline has the most comprehensive literature review, with an extensive text summary of the treatment literature and an exhaustive list of references. But bulk comes as an unavoidable side effect of comprehensiveness. Though the IPS guidelines are less comprehensive than the APA guidelines, yet they address almost all the important areas of any illness. The literature review is also comprehensive, and attempt has been made to include almost all possible significant studies. The IPS guidelines have been carefully drafted to limit the bulk, even after including the maximum needed information. The PORT guideline references detailed review articles, whereas the Expert Consensus guidelines and the TMAP algorithms include only limited literature reviews and selected references.

### Number and depth of recommendations

The APA and Expert Consensus guidelines make the most specific treatment recommendations, and PORT makes the fewest. Only a minority of the APA and PORT recommendations are contingent on a patient's treatment history. In contrast, the recommendations listed in the TMAP algorithms are based exclusively on the patient's treatment and response history. The IPS guidelines make recommendations on the basis of history, as well as mental status examination, and also address special issues elaborately. The recommendations made are clear and specific; the strength with which the recommendations are made is lacking.

### Phases of the illness

The IPS, APA, PORT and Expert Consensus guidelines include specific recommendations about treatment of the acute, continuation/stabilization, maintenance/stable phase of various mental illnesses. TMAP indicates that its algorithms are suitable for “any patient beginning medication treatment or taking a medication without a satisfactory response.”

### Content

The IPS and APA guidelines include several recommendations about pharmacological management, group and individual therapy, vocational rehabilitation and specific psychosocial interventions. The PORT recommendations focus mainly on pharmacotherapy; but recommendations are also made for family interventions, psychological interventions, vocational rehabilitation and case management. The Expert Consensus guidelines address both pharmacological and psychosocial interventions and offer recommendations about the intensity or location of nonpharmacological services. In contrast, the TMAP algorithms address only the pharmacological management of schizophrenia.

### Costs

Cost-benefit ratio is one of the prime concerns of a clinician, especially while practicing in a developing country like ours. Developers of Indian guidelines failed to conceptualize this important aspect in the IPS guidelines. There is no mention of the cost of therapy of any of the illnesses; and thus at places, the recommendations have to be undermined while practicing. In fact, none of these guidelines include specific considerations about costs and resource allocation in their recommendations. Although the APA guidelines note that the advantages of the newer antipsychotic medications should be balanced against their costs, the PORT recommendations refer to the costs of assertive community treatment and clozapine; and the Expert Consensus guidelines include a policy section that recommends interventions to increase the cost-effectiveness of mental-health services.

## CLINICAL APPLICABILITY

### Ease of use

The algorithmic format employed by TMAP makes it the easiest to use. The IPS guidelines also have the distinct advantage of being easy to use and comprehend. The guidelines clearly convey the duration of treatment and other important issues. The algorithms given at the end of chapters further simplify the user's understanding. In the text also, rather than using a “paragraph” format, a point-wise listing of the guidelines makes them easier to comprehend and follow. The Expert Consensus guidelines, designed to be user-friendly, list recommendations in tabular form. The numbered PORT recommendations are quickly assimilated. The APA guidelines, written in the form of a literature review, are more difficult to assimilate and use. Almost all the guidelines barring the TMAP repeat the information given under various headings, making them unnecessarily cumbersome.

### Applicability in the local setting

A major difference between the IPS guidelines and other international guidelines is the area targeted by the guidelines. While the IPS guidelines are supposed to serve as guiding principles for the Indian psychiatrists, the other guidelines are more international in their approach. This fact has not been properly reflected in the drafting of the IPS guidelines. Mentions have been made of drugs or psychotherapeutic techniques which are not available in India or are available at very few centers. The guidelines do not give enough emphasis to the available resources while making the recommendations.

### Timely review

The APA guidelines, published in 1997, were based on reviews of the scientific literature up to 1995. Revisions are scheduled at three- to five-year intervals. The PORT was based on literature reviews up to 1993 and no revisions are scheduled. The Expert Consensus guidelines were originally published in 1996 and were updated in 1999. TMAP revisions are made available at the TMAP website as new antipsychotic agents are introduced; the latest revision occurred in December 1999. The IPS guidelines are pretty young and a review can wait for another year or so, but timely review should be accepted as a policy.

### Specificity or ease of operationalizing

Guidelines that are specific and can be operationalized as rate-based monitors lend themselves to benchmarking and the establishment of best-practice parameters. Both the TMAP algorithms and the PORT recommendations are explicit, specific and easily operationalized. The Expert Consensus guidelines are more general; and the IPS and APA guidelines are almost didactic in format, making them more difficult to operationalize.

## SHORTCOMINGS OF IPS PRACTICE GUIDELINES

In order to improve further, it is necessary to identify one's weaknesses. Therefore, just to aid in future improvements and not with any intention of criticism, we are mentioning a few areas needing improvement in the days to come.

The development process of the guidelines should be more defined so as to allow a clinician to choose a recommendation based on its merit. The researchers across the country should be urged to contribute more for the development of the guidelines and in turn psychiatry as a whole, but efforts will be needed to minimize the contributor bias. The guidelines have been formulated with the intention of being universal and thus bringing accountability to the practice of Psychiatry in India. This universal appeal will need active participation not only of the prominent psychiatrists in India but also from small-town mental-health professionals. Though inputs are always welcome, they must be included only after carefully weighing the scientific emphasis they bring along. All the included studies must be assessed for their scientific legitimacy and quality, before making a recommendation based on the studies. Only enumeration of the studies might not be sufficient, but guidelines based on the regional researches will be more helpful.

The APA guidelines give us an important example of rating the recommendations. By doing this they clearly show the strength with which they make a particular recommendation, which makes it much easier for the clinician to make decisions.

The IPS guidelines lack a structured format which must be rigidly followed for describing all the illnesses, making the guidelines more uniform.

Cost and resource considerations are one of the prime concerns of a clinician while prescribing treatment in a developing country like ours; unfortunately, this important issue has not been adequately addressed in the Indian guidelines. This aspect becomes more important as the insurance coverage is not extensive in India, unlike Western nations.

Apart from cost, psychosocial and cultural considerations are also very important in our setup, especially while guiding the practitioner for nonpharmacological management. The strong family and social support systems in our country make the needs different, and also the unavailability of experts and infrastructure limits our armamentarium of psychotherapies.

Dosages for medications have been found to be varying with race or ethnicity. Specific recommendations made for dosages of antipsychotics and other medications might be needed to suit the Indian population.

Lastly, the guidelines can be made more user-friendly and easy to use by trying to be more precise and avoiding repetition of information.

## DISCUSSION

An ideal guideline for clinical practice would combine the best features of each of these efforts. The development process would be defined and specified in the guideline. It would be derived from a comprehensive literature review. It would explicitly assess the quality of supporting research studies and the methods used for synthesizing evidence. Evidence tables would be constructed and published in an ideal guideline. Questionnaires can be prepared to receive input from mental-health professionals all over the country. Both evidence-based and expert opinions need to be incorporated, but with a clear delineation between the two. Treatment recommendations would take into account important demographic, cultural and psychosocial issues. Cost considerations would be included. Dosage requirements for Indian population would be worked out and incorporated in the guidelines. Recommendations would be summarized in a concise, user-friendly format; and suggestions would be made about how the guidelines might be operationalized as quality monitors. Guidelines would be revised at three-year intervals.

Guidelines that address the factors described in this review may be more effective in optimizing clinical practice. However, even excellent guidelines will require additional support, such as academic detailing and provider reminders, if best practices are to be disseminated widely. Though we strive to define a universal and ideal guideline, the importance of individual knowledge and belief can never be undermined.

The standardized must gel with the personalized and the socialized to make for good psychiatric practice. It is a moot point whether a nomothetic-idiographic orientation is valid only for Psychiatry or for all of medicine.[6]

## References

[1] McIntyre JS, Charles SC (2004). American Psychiatric Association Practice guidelines for the treatment of psychiatric disorders. Compendium.

[2] Lehman AF, Steinwachs DM (1998). Translating research into practice: The Schizophrenia Patient Outcomes Research Team (PORT) treatment recommendations. Schizophr Bull.

[3] McEvoy JP, Scheifler PL, Frances A (1999). The Expert Consensus Guideline Series: Treatment of Schizophrenia. J Clin Psychiatry.

[4] Miller AL, Hall CS, Crismon ML, Chiles JA (2003). Texas Implementation of Medication Algorithm. procedural manual- Schizophrenia Module.

[5] Gautam A, Awasthi A, Trivedi JK, Kulhara P, Shah N, IPS Task Force (2004). other members of the committee. Clinical Practice Guidelines for Psychiatrists in India.

[6] Singh A, Singh S (2005). (2004) Resolution of the Polarization of Ideologies and Approaches in Psychiatry, Mens Sana Monographs (2004-2005), II: 4-5, Nov 2004.

